# A scoping review of implementation science theories, models, and frameworks — an appraisal of purpose, characteristics, usability, applicability, and testability

**DOI:** 10.1186/s13012-023-01296-x

**Published:** 2023-09-19

**Authors:** Yingxuan Wang, Eliza Lai-Yi Wong, Per Nilsen, Vincent Chi-ho Chung, Yue Tian, Eng-Kiong Yeoh

**Affiliations:** 1 JC School of Public Health and Primary Care, Faculty of Medicine, The Chinese University of Hong Kong, Shatin, Hong Kong China; 2 Centre for Health Systems and Policy Research, JC School of Public Health and Primary Care, Faculty of Medicine, The Chinese University of Hong Kong, Shatin, Hong Kong China; 3 Division of Community Medicine, Department of Medical and Health Sciences, Linköping University, 581 83 Linköping, Sweden; 4 School of Chinese Medicine, The Chinese University of Hong Kong, Shatin, Hong Kong China

**Keywords:** Implementation science, Implementation research, Theories, Models, Frameworks, Diffusion, Dissemination, Knowledge translation

## Abstract

**Background:**

A proliferation of theories, models, and frameworks (TMFs) have been developed in the implementation science field to facilitate the implementation process. The basic features of these TMFs have been identified by several reviews. However, systematic appraisals on the quality of these TMFs are inadequate. To fill this gap, this study aimed to assess the usability, applicability, and testability of the current TMFs in a structured way.

**Methods:**

A scoping review method was employed. Electronic databases were searched to locate English and Chinese articles published between January 2000 and April 2022. Search terms were specific to implementation science. Additionally, hand searches were administered to identify articles from related reviews. Purpose and characteristics such as the type of TMF, analytical level, and observation unit were extracted. Structured appraisal criteria were adapted from Birken et al.’s Theory Comparison and Selection Tool (T-CaST) to conduct an in-depth analysis of the TMFs’ usability, applicability, and testability.

**Results:**

A total of 143 TMFs were included in this analysis. Among them, the most common purpose was to identify barriers and facilitators. Most TMFs applied the descriptive method to summarize the included constructs or the prescriptive method to propose courses of implementation actions. TMFs were mainly mid-range theories built on existing conceptual frameworks or demonstrated grand theories. The usability of the TMFs needs to be improved in terms of the provision of conceptually matched strategies to barriers and facilitators and instructions on the TMFs usage. Regarding the applicability, little attention was paid to the constructs of macro-level context, stages of scale-up and sustainability, and implementation outcomes like feasibility, cost, and penetration. Also, fewer TMFs could propose recommended research and measurement methods to apply the TMFs. Lastly, explicit hypotheses or propositions were lacking in most of the TMFs, and empirical evidence was lacking to support the claimed mechanisms between framework elements in testability.

**Conclusions:**

Common limitations were found in the usability, application, and testability of the current TMFs. The findings of this review could provide insights for developers of TMFs for future theoretical advancements.

**Supplementary Information:**

The online version contains supplementary material available at 10.1186/s13012-023-01296-x.

Contributions to the literature

- This scoping review fills the research gap on the quality of TMFs being developed and applied in the implementation science field during the proliferation of theories, Models, and frameworks (TMFs).
- The findings of this review contribute to the conceptual development of implementation science by systematically appraising the usability, applicability, and testability of TMFs, which could provide insights for TMF developers.
- This scoping review provides an updated comprehensive list of current TMFs in the implementation science field.

## Background

Implementation science was developed to address the challenges of adopting research and evidence-based practices (EBPs) into routine practice, improving the quality and effectiveness of health services [1, 2]. After two decades of development, this field has shifted from being empirically driven to an emphasis on using theories, models, and frameworks (TMFs) [2] due to the increased recognition of their importance for understanding and explaining complex implementation processes and outcomes [3].

TMFs have been shown to promote generalization by offering common language and constructs, therefore facilitating communication and shared understanding [4]. TMFs are also prominent in guiding implementation planning, understanding influential factors of successful implementation, and selecting implementation strategies [5]. With the potential benefits of conceptual development [6], the number of TMFs continues to grow [7, 8]. However, the proliferation of TMFs may impede the process of identifying and selecting the most suitable ones to support implementation projects [9]. Research has also indicated that the misuse or superficial use of TMFs is not uncommon [4].

Several reviews have summarized the characteristics of existing TMFs to understand the current theoretical scope and aid the selection of TMFs for practitioners [7, 10–13]. Tabak et al. conducted a narrative review of 61 different TMFs to identify and examine the currently used models in the dissemination and implementation field [7]. A second narrative review searched articles from 1990 to 2014 and found 41 different research translation frameworks and models [10]. A scoping review by Strifter et al. identified 159 TMFs from 596 studies, but the main focus was the application of TMFs in chronic disease and cancer research [11]. Recently, another scoping review built on Strifter et al. aimed to provide a list of full-spectrum TMFs, covering all phases of knowledge translation for researchers to choose from [12]. Furthermore, a systematic review by Moullin et al. was conducted to assess the comprehensiveness of 49 TMFs by examining the degree and depth of analysis; however, the criteria were relatively arbitrary [13]. Overall, the reviews have used different concepts such as knowledge translation, research translation, or dissemination and implementation. Although these concepts are often used interchangeably [12], they are not synonymous [14]. The data extraction items were similar and somewhat elementary among these reviews. Moreover, most of the reviews focused on the characteristics of TMFs but paid little attention to the systematic quality appraisal of the TMFs. Undesirable implementation outcomes may occur if TMFs are challenging to use, apply, and test. Therefore, it is necessary to generate a deeper understanding beyond the descriptive summarization of the characteristics for implementation scientists to promote the scientific development of this field.

In summary, conducting a rigorous quality assessment is vital to improve the scientific soundness of TMFs further and maximize their impact. As a result, this review will serve as a reference for researchers to generate new TMFs and refine the current TMFs by identifying the limitations of the existing TMFs. Thus, this scoping review aims to critically review the purposes and characteristics of the TMFs that are explicitly described in the implementation science field, but also appraise the quality of the TMFs by employing a reliable scale, which can evaluate the theories on usability, applicability, and testability developed by Birken et al. [4]. Appraisal of the quality of TMFs in implementation science is essential to enhance the understanding and explanation of the implementation processes and outcomes in this still-developing field.

## Method

We developed this study according to the scoping review methods by Arksey and O’Malley [15]. The Preferred Reporting Items for Systematic Review and Meta-Analysis extension for Scoping Review (PRISMA-ScR) guideline was followed in the reporting of our results [16].

### Search strategy

To identify TMFs related to implementation science, we researched multiple electronic databases, including MEDLINE, Embase, PsycINFO, Global Health, PubMed, and Web of Science. The search was limited to English articles published between January 2000 and April 2022. The search strings were "implementation science" or "implementation research" and "model* or theor* or framework*" to target a specified range of articles with the subject of implementation science TMFs. In addition, hand searches were administered according to TMF reviews [7, 10–13]. We also searched Chinese articles on Chinese databases, including CNKI, CCPD, and CSCD, and the Chinese Social Science Citation Index, published from inception until April 2022. The search strings were the same as the English terms translated into Chinese.

### Inclusion/exclusion criteria

Studies were selected if they met the inclusion criteria as follows:

1. Articles published in English or Chinese
2. *Implementation science* was defined as “the scientific study of methods to promote the systematic uptake of research findings and other evidence-based practices into routine practice, and hence, to improve the quality and effectiveness of health service” [1].
3. *Framework* was defined as the “structure, overview outline, system or plan consisting of various descriptive categories” [2].
4. *Model* was defined as “a deliberate simplification of a phenomenon on a specific aspect of a phenomenon” [2].
5. *Theory* was defined as “a set of analytical principles or statements designed to structure our observation, understanding, and explanation of the world” [2].
6. Proposed new TMFs or modified an existing TMF.
7. Applicable to public health or healthcare disciplines.

Studies were excluded if they were as follows:

1. Studies where no TMF was proposed or mentioned
2. Reported an existing TMF without any modification
3. Program theory based on a single case study
4. Study protocols
5. Conference abstracts
6. Thesis

### Data collection

A single reviewer (Y. X. W.) reviewed the title and abstract. Full-text articles were then obtained and assessed based on the inclusion criteria. TMFs developed without evidence-based practices were excluded. Articles with proposed structures that could not be defined as frameworks or models or articles with proposed statements that did not meet the definition of theory were also excluded. Articles that the first reviewer was uncertain about were discussed with a professional of the respective field in our research team (ELYW) to come to a consensus for the inclusion or exclusion decisions.

### Data extraction

Data were extracted by the same reviewer (Y. X. W.). A second reviewer (Y. T.) randomly selected 10% of the articles and did the data extraction. Discrepancies were discussed and resolved with mutual consensus. The final results were further reviewed by professionals in this field (N. P. and E. L. Y. W.). Items that were extracted from the studies were as follows:

1. The purpose of the TMFs: A coding scheme adapted from Birken et al. [5] was adopted as a priori to do the abstraction. Nine purposes were used to capture all the potential purposes of the development of the TMFs. We set no limit to new emerging purposes, which would be added to the coding scheme during the review process.
2. Characteristics of the TMFs: Four items were extracted:
  1. The category of the TMFs adapted from Nilsen (determinant framework, classic theory, implementation theory, evaluation framework, and process model) [2]. Strategy framework, defined as the structure of the implementation interventions to facilitate the implementation process, and measurement framework, defined as the structure of the measurement metrics of implementation constructs or influential factors, were added to the original TMF category to represent the newfound function of the TMFs.
  2. The theoretical underpinning of the TMFs
  3. The theory level of the TMFs, which was adapted from Kislov et al. (grand theory, mid-range theory, and program theory) [3]
  4. The level of analysis of the TMFs adapted from the business analytics field (descriptive, diagnostic, predictive, and prescriptive) [17]

The detailed definitions can be found in Additional file 1 [RM1].

3. The usability of the TMFs: Five metrics were used to assess the usability. These criteria were adapted from a TMF selection tool called T-CaST developed by Birken et al. [4]. This tool was chosen because it was developed particularly for implementation science TMFs. It was also face-validated by endorsing the opinions of 37 professionals with ample experience in implementation science. There are six original metrics under the “usability” domain according to this tool:
  1. Relevancy of the constructs: The authors explain the relevancy of each construct of the TMF.
  2. Diagram of the TMFs: The proposed TMF has a clear and useful figure depicting the included constructs and relationships among them.
  3. Guidance for application: The authors provide a step-by-step approach for applying the TMF.
  4. Change strategy: The TMF provides methods for promoting the implementation in practice
  5. Mechanism and relationships between the constructs: The TMF provides an explanation of how the included constructs influence the implementation and each other

The sixth appraisal item, “Key stakeholders are able to understand, apply, and operationalize the TMF,” was excluded because it was considered subjective and required the applicants’ own discretion.

4. Applicability of the TMFs: We adjusted the original five metrics under the “applicability” domain of the T-CaST tool [4] to accommodate this study’s analysis. There were five metrics after the revision:
  1. TMFs focus on a relevant implementation science theme, adapted from the “TMF focuses on a relevant implementation outcome.” Six themes were studied according to Nilsen et al. [18]: “context,” “strategies,” “outcomes,” “fidelity,” “adaptation,” and “sustainability.” We also added “process” as another theme to represent the TMFs concerning the implementation process.
  2. Proposed research and measurement methods: The authors provide a particular method of research that can be used with the TMF, such as interviews, surveys, and chart reviews.
  3. Level of change: The TMF addresses a relevant analytic level.
  4. Generalizability: The TMF is generalizable to other disciplines, settings, and populations.
  5. Innovation type: The TMF is designed for a specific type of innovation, and the classifications, adapted from Moullin et al.’s review [13], are intervention, guideline, knowledge, policy, and implementation programs

The metric of “the TMF has been used in a relevant population” was eliminated because we included this information when analyzing the “generalizability” of each TMF by articulating whether the TMF targeted a specific population.

5. Testability of TMFs: We employed all three metrics under the “testability” domain of the T-CaST tool [4]:
  1. Proposed an explicit hypothesis, assumptions, or propositions.
  2. Evidence of change mechanism: The TMF includes meaningful, face-valid evidence of the proposed relationship.
  3. Empirical support: The TMF contributes to the evidence base and has been used in empirical studies.

The domain of “acceptability” under the T-CaST tool was excluded from this study. The first metric, “TMF is familiar to key stakeholders,” was considered subjective; the second metric, “TMF comes from a particular discipline,” was not applicable to this study because we aimed to focus on the healthcare field only. Therefore, the final version of the appraisal criteria used by this study consisted of three domains (usability, applicability, and testability) and included a total of 13 metrics. The definition of each metric is elaborated on in Additional file 1.

Considering that the primary purpose of this study was to understand the current development and variety of TMFs in the implementation science field, quality appraisals of the studies would be the next step for future research.

### Synthesis of results

TMFs were initially categorized based on the purpose of the TMF and its characteristics. Critical analyses were then performed and presented in the form of the percentage and counting numbers of TMFs according to each item of the appraisal metrics.

In order to investigate the overall quality of the TMFs included in addition to the descriptive analysis of each metric, we assigned scores to quantify the 13 metrics to see how many TMFs met the “high-quality” standard and which domains (usability, applicability, or testability) needed further improvement. The evaluation was quantified by assigning a score of one if the TMF met the specific criterion and zero if it did not. Among the 13 measured metrics, 10 metrics with a “yes or no” answer could be quantified. The details are found in Additional file 1. During the quantification process, we initially performed a horizontal analysis to appraise each of the TMFs concerning the ten metrics; the score range was 0–10. We used the priori that TMFs scoring 7 and above would be considered high quality. A vertical analysis was then performed by looking at the data of each metric and domain, crossing all included TMFs. The total score was standardized to 100, and the range was 0–100. A narrative description and tabular format were applied to incorporate and organize the data in the final results.

## Results

### Search results

**Fig. 1:**
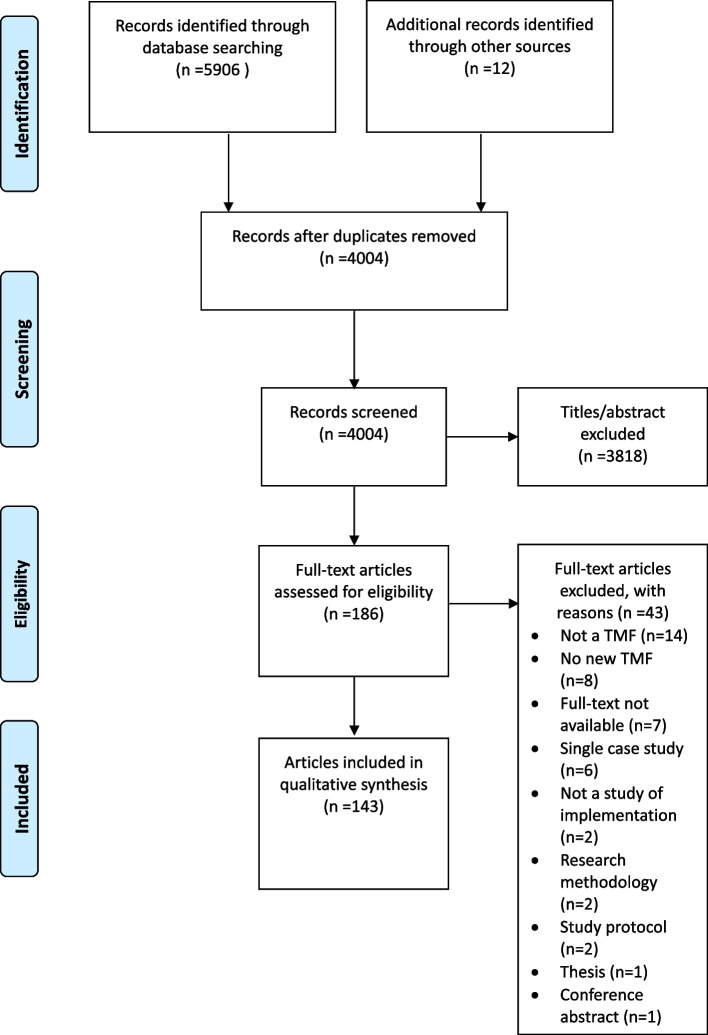
PRISMA flow chart of framework selection

The database search yielded 5906 articles; hand searches identified another 36 articles. A total of 4004 articles were left after removing duplicates. After title and abstract screening, 186 articles were included in the full-text review. During the full-text review, 43 articles were excluded: 14 articles did not mention a TMF, 8 articles did not propose a new TMF, 7 articles did not have full text, 6 articles were single case program theories, 2 articles were not related to implementation, and the remaining 6 articles were not drafted as research articles. Therefore, 143 articles describing 143 different TMFs were included for data analysis (Fig. 1).

### Purpose and characteristics of TMFs

Of the 143 TMFs, 52 (36%) were developed to identify barriers and facilitators (e.g., Theoretical Domains Framework) [19], 18 (13%) were intended to guide the design or selection of implementation strategies (e.g., behavior change wheel) [20], and another 19 (14%) specified the process of implementation (e.g., KTA model) [21]. Seventeen (12%) TMFs aimed to frame the evaluation (e.g., RE-AIM) [22], and another 17 aimed to guide implementation planning (e.g., i-PARIHS) [23]. Eleven of the 143 (8%) TMFs were primarily conducted to enhance conceptual clarity (e.g., community-integrated intermediary) [24]. There were relatively few studies aimed at specifying relationships between constructs (*n* = 5; 3%) (e.g., system change framework) [25], informing data analysis (*n* = 2; 1%) (e.g., stakeholder analysis) [26], and data collection (*n* = 2; 1%) (e.g., FRAME) [27].

Among the 143 TMFs, the most common category of TMF was determinant frameworks, which accounted for 64 out of the 143 (45%) (e.g., Roger’s framework) [28] followed by 36 (25%) process models (e.g., Iowa model) [29], 31 (22%) strategy frameworks (e.g., ARC model) [30], 27 (19%) evaluation frameworks (e.g., HOT-fit framework) [31], and the remaining 5 were measurement frameworks (e.g., organizational readiness for implementing change) [32].

We made a post hoc analysis on the relationship between the purpose and the categorization of the TMFs (Table 1). Determinant frameworks were the most frequently used to identify barriers and facilitators (*n* = 52; 81%), and evaluation frameworks were common for framing the evaluation, including formative and summative evaluations (*n* = 17; 63%), strategy frameworks are helpful to guide strategy design (*n* = 16; 52%), and process models are mainly for guiding implementation planning (*n* = 12; 34%) and specifying the process of implementation (*n* = 19; 53%).

**Table 1:** Purpose by TMFs category

Purpose, *n* (%)	TMFs category				
	**Determinant framework**	**Evaluation framework**	**Strategy framework**	**Measurement framework**	**Process model**
Enhance conceptual clarity	4 (6)	2 (7)	6 (19)	0 (0)	0 (0)
Frame evaluation	1 (2)	17 (63)	0 (0)	1 (20)	2 (6)
Guide design or selection of IS strategies	3 (5)	1 (4)	16 (52)	0 (0)	2 (6)
Guide implementation planning	2 (3)	1 (4)	4 (13)	0 (0)	12 (33)
Identify barriers and facilitators	52 (81)	3 (11)	0 (0)	2 (40)	1 (3)
Inform data analysis	1 (2)	0 (0)	0 (0)	2 (40)	0 (0)
Inform data collection	0 (0)	2 (7)	0 (0)	0 (0)	0 (0)
Specify process of implementation	0 (0)	0 (0)	2 (6)	0 (0)	19 (53)
Specify relationship between constructs Total	1 (2) 64 (100)	1 (4) 27 (100)	3 (10) 31 (100)	0 (0) 5 (100)	0 (0) 36 (100)

In terms of the theoretical underpinning, 51 (36%) of the TMFs were supported by one or more grand theories, such as social science theories, behavioral science theories, or information science theories, and 79 (55%) were built on one or more existing TMFs, such as the consolidated framework for implementation research (CFIR) [33], diffusion of innovation [34], or technology acceptance model [35].

Most of the TMFs were identified as mid-range theories (*n* = 103) (e.g., general theory of implementation) [36], 17 were defined as high-order program theories (e.g., QUERI Impact Framework) [37], and three grand theories were identified in this review [38–40].

Regarding the analytics, most of the TMFs fell into descriptive frameworks (*n* = 57; 40%) (e.g., CFIR) [19], 42 (29%) were categorized as prescriptive frameworks (e.g., K2A model) [41], 31 (22%) were identified as diagnostic frameworks (e.g., MADI model) [42], and 13 (9%) were predictive TMFs (e.g., CASCADA theory) [43].

### Usability appraisal of the TMFs

Five metrics were used to assess the usability of the TMFs (Table 2). Most TMFs explained the relevancy of the included constructs (*n* = 133; 93%). Over half of the TMFs (*n* = 84; 59%) provided step-by-step guidance for applying the TMF, and a large amount of the TMFs (*n* = 133; 93%) provided a diagram or table to show or explain the constructs. Regarding change strategies provided, 71 TMFs (59%) met the criteria, and 22 evaluation frameworks did not apply to this criterion since they aimed to guide evaluations instead of facilitating the implementation process. Seventy-five (71%) TMFs included descriptions of the change mechanism or the relationship between constructs, and 37 TMFs under the process model were labeled as not applicable since the primary focus of this group was not related to identifying impact factors or underlying relationships.

**Table 2:** Usability appraisal of TMFs

	**Number of TMFs (*n* = 143)**	**Example TMFs**
**Relevancy of constructs, *n* (%)**		
Y	133 (93)	[43–45]
N	10 (7)	-
NA	0	-
**Diagram of TMFs, *n* (%)**		
Y	133 (93)	[46–48]
N	10 (7)	-
NA	0	-
**Guidance for application, *n* (%)**		
Y	84 (59)	[30, 49, 50]
N	59 (41)	-
NA	0	-
**Change strategies, *n* (%)**		
Y	71 (59)	[51–53]
N	50 (41)	-
NA	22	-
**Mechanism/relationships between constructs, *n* (%)**		
Y	75 (71)	[36, 54, 55]
N	31 (29)	-
NA	37	-

### Applicability appraisal of the TMFs

Five metrics were employed to appraise the applicability of the TMFs (Table 3).

- *Implementation science constructs*: “Context” (*n* = 87), “process” (*n* = 77), and “strategy” (*n* = 70) were the most frequently studied constructs, which accounted for 61%, 54%, and 49% of the TMFs, respectively, while a relatively small amount of TMFs focused on “outcome” (*n* = 34; 24%), “fidelity/adaptation” (*n* = 11; 8%), and “sustainability” (*n* = 6; 4%).
- *Research/measurement method*: Over half of the TMFs (*n* = 87; 61%) were neither providing a measurable scale nor recommending a research method, but there were still 39% of the TMFs that either proposed possible methods, such as interviews, questionnaires, hybrid design, and comparative studies that could be employed or made a reference to the available scales or self-designed scales to measure the construct.
- *Analysis level*: The most common level of analysis was identified as full spectrum (*n* = 57; 40%), followed by individual level (*n* = 47; 33%), intervention level (*n* = 36; 25%), and organizational level (*n* = 33, 23%). However, levels like system (*n* = 17; 12%), team (*n* = 7; 5%), and policy (*n* = 3; 2%) were seldom addressed.
- *Generalizability*: Generalizable TMFs could fit into multiple situations without specific context requirements, such as settings and target audiences. As for the implementation setting, 105 TMFs (73%) were not developed for a specific physical environment. Fifteen TMFs (10%) articulated that the community would be the ideal application setting; among them, one targeted the disadvantaged community [51]. Fifteen TMFs (10%) were designed for organizational use. There were also four TMFs (3%) aiming to facilitate the implementation research in low- and middle-income countries. Innovation target audiences were mostly not specified by the TMF developers (*n* = 125; 87%), but several TMFs were designed for a specific group of people, such as clinical practitioners (*n* = 6), vulnerable people (*n* = 5), children and families (*n* = 3), and elders (*n* = 1). Based on this information, 87 TMFs (61%) were found to be generalizable, and 56 (39%) were grouped as being individual.
- *Innovation type*: Interventions were the most studied innovation type, including interventions, programs, innovations, complex innovations, shared-decision making, technologies, evidence-based practices, telehealth, service, QI project, and integrated care (*n* = 95), followed by implementation programs (*n* = 15); guidelines, including clinical practice, best practice, guideline, deprescribing, and process (*n* = 10); knowledge, including knowledge, research, and ethical norms (*n* = 10); and policy (*n* = 5).

**Table 3:** Applicability appraisal of TMFs

	**Number of TMFs (*n* = 143)**	**Example TMFs**
**TMF covers related constructs, *n* (%)**		
Context	87 (61)	[19, 56, 57]
Process	77 (54)	[22, 58, 59]
Strategy	70 (49)	[41, 60, 61]
Outcome	34 (24)	[62–64]
Fidelity/adaptation	11 (8)	[65–67]
Sustainability	6 (4)	[68–70]
**Proposed research/measurement method, *n* (%)**		
No	87 (61)	-
Yes	55 (39)	[48, 71, 72]
NA	1	-
**Analytic level, *n* (%)**		
Full spectrum	57 (40)	[27, 73, 74]
Individual	47 (33)	[19, 20, 43]
Intervention	36 (25)	[31, 75, 76]
Organization	33 (23)	[77–79]
System/social	17 (12)	[54, 80, 81]
Team	7 (5)	[82–84]
Community	4 (3)	[30, 53, 78, 85]
Policy	3 (2)	[20, 45, 86]
Cross-national	1 (1)	[87]
**Generalizability, *n* (%)**		
Yes	87 (61)	[88–90]
No	56 (39)	-
**Implementation setting, *n* (%)**		
Not specified	105 (73)	-
Community	15 (10)	[59, 91, 92]
Organization	15 (10)	[79, 93, 94]
LMIC	4 (3)	[26, 95–97]
Global	2 (1)	[87, 98]
Public service sector	2 (1)	[57, 86]
**Target audience, *n* (%)**		
Not specified	125 (87)	-
Clinical practitioner	6 (4)	[48, 75, 99]
Vulnerable population	5 (4)	[73, 91, 100–102]
Children and/or families	3 (2)	[30, 57, 103]
Discharged patients	1 (1)	[44]
Elders	1 (1)	[92]
Indigenous population	1 (1)	[53]
Adolescents and adults	1 (1)	[67]
**Innovation type, *n* (%)**		
Intervention	95 (66)	[74, 104, 105]
Implementation program	14 (10)	[46, 106, 107]
Knowledge	11 (8)	[108–110]
Guideline	10 (7)	[63, 84, 111]
Not specified	8 (6)	-
Policy	5 (3)	[24, 26, 45, 62, 86]

### Testability appraisal of the TMFs

Three criteria were employed to assess the testability of the TMFs (Table 4).

- *Proposed explicit hypothesis, assumptions, or propositions*: The first metric was concerned with whether an explicit hypothesis was proposed in the article. However, we could not find an explicit hypothesis, assumption, or proposition to be tested in most of the articles (*n* = 115; 80%).
- *Evidence of change mechanism provided*: Out of the 75 papers describing change mechanisms, we found that only 43 (57%) could provide sound evidence to support the claims.
- *Empirical support*: The last metric was whether the TMFs had empirical support; we found that three-quarters (*n* = 108; 76%) of the TMFs were qualified for this metric. We further identified the type of evidence and found that the two most common empirical evidence were case studies (*n* = 39) and literature reviews, including systematic and scoping reviews (*n* = 49). Case studies were mainly used for TMF refinement after the initial draft had been formulated, while literature reviews were often employed at the development stage of the TMFs to generate the evidence pool.

**Table 4:** Testability appraisal of TMFs

	**Number of TMFs (*n* = 143)**	**Example TMFs**
**Explicit hypothesis, *n* (%)**		
Y	27 (20)	[42, 112, 113]
N	115 (80)	-
NA	1	-
**Evidence of change mechanism, *n* (%)**		
Y	43 (57)	[51, 114, 115]
N	32 (43)	-
NA	68	-
**Empirical support, *n* (%)**		
Y	108 (76)	[47, 55, 116]
N	35 (24)	-
NA	0	-

### Quantification analysis

The horizontal analysis showed that one-third of the TMFs (*n* = 49; 34%) scored a 7 or above (score range was 0–10), which met the high-quality standard. Over half of the TMFs (53%) scored between a 5 and 7, and the remaining 13% scored below a five. No TMFs scored below a 3. In addition, the vertical analysis showed that the domain of “usability” had the highest score (75/100) compared with applicability (61/100) and testability (47/100). In terms of specific metrics, two metrics, “explanation of construct” and “diagram presented” under “usability,” scored the highest (93/100), while “propose a testable hypothesis” under “testability” scored the lowest (19/100) followed by “propose research/measurement method” under “applicability” (39/100).

## Discussion

### Key findings

This review identified 143 TMFs. The large number reflects an increasing focus on this field’s theoretical and conceptual development. Our study summarized the purpose and characteristics of the TMFs; critically assessed the TMFs on usability, applicability, and testability; and quantified the overall quality. To our knowledge, this is the first scoping review to provide a structured appraisal to enhance the understanding of theoretical development, identify limitations, and facilitate the refinement of TMFs empirically.

Among the TMFs, determinant frameworks were the most common, while measurement frameworks were the least common. Although some of the determinant frameworks were accompanied by measurement methods, many context factors could not be measured with credible scales. According to Chaudoir et al.’s review, measures of structural-level and patient-level determinants were scarce, and most measurement scales were without proper examination of criterion validity [117]. However, criterion validity issues are crucial regarding the development of the implementation science field because they could inform the refinement of implementation science theories by examining the hypothesized relations among constructs proposed by TMFs [118]. In addition, researchers found that different measurement scales were used to measure similar implementation constructs, which could impede the comparison of evidence [118]. It is important for field development to standardize the implementation construct concepts and align measures and constructs. Therefore, future research is needed to develop reliable and specific scales of relevant contextual factors addressed by those determinant frameworks.

Theory underpinning helps to ensure the internal coherence and logically sound structure TMFs. Most of the TMFs were derived from one or more grand theories of different disciplines. This convergence may imply that implementation is a complex subject with multiple contexts and interactions [119]. Insights from other disciplines may be helpful to enhance the explanation and prompt new theory development in this field [120].

TMFs classified as mid-range theories were the most common. According to Kislov et al., mid-range theories not only link empirical observations and high-level abstract views of grand theories but also guide program theory development with their potential to generate propositions and hypotheses [3]. Reciprocally, empirical evidence derived from program theories could test and refine the mid-range theories and stimulate new knowledge of grand theories. Thus, researchers are suggested to employ a longitudinal design to examine the causal effects via the appliance of the mid-range theories.

According to our results, most of the TMFs were descriptive, narrating the characteristics of the constructs, which was consistent with Moullin et al.’s review [13]. Prescriptive frameworks were significantly more extensive compared with Moullin et al.’s study, implying that process guidance was more focused in this field. However, consistent with the same review, predictive frameworks were still scarce. This was probably because most frameworks were designed retrospectively based on the developers’ previous experiences or multiple case studies. Researchers were more likely to generate diagnostic or explanatory frameworks instead of predictions.

With regard to the usability assessment, most authors could well explain the relevancy of each element, component, or construct of their proposed TMFs. Clear descriptions of the constitution were necessary to facilitate their use [121]. However, determinant frameworks were seldom linked to paired strategies to overcome barriers and enhance facilitators. Implementation scientists have generated several comprehensive strategy taxonomies for implementation scientists to choose from, such as ERIC [122, 123] and EPOC [124], but to what extent specific strategies could address determinants were not tested [125]. A previous systematic review aimed to find evidence of the mechanism of strategies to understand how and why they work; however, the authors found that just one-third of the included studies examined the mechanism of the strategy, and the lack of high-quality studies impeded synthesizing the findings across studies to generate promising mechanisms [126]. Additionally, only 60% of the TMFs could provide clear directions for their application. However, this is important as it could serve as a mediation to transform abstract TMFs into practical and concrete tasks, especially for novice practitioners.

After performing the applicability assessment, we found that research on concepts of “adaptation,” fidelity,” and “sustainability” was less common. Adaptation and its relationship with fidelity are imperative to successful implementation because dynamic implementation and adaptation may frequently occur for various purposes [127]. The ability to adapt was also recognized as one of the requirements of sustainability [128]. Promoting the long-term impact of the innovations would be significant to stakeholders and health planners for avoiding unnecessary waste [129]. Further studies are warranted on these emerging concepts to enhance the comprehensiveness of this field.

We found that TMFs studying “strategies” were usually included in a set of activities. Practitioners could follow specific activities to administer the implementation projects. However, limited information was provided on the weights, impact, and chronological order of these activities. The interactions between activities were also not well tested. This is consistent with a previous review that showed unclear attribution of these recommended activities had affected the successful implementation [8]. Empirical evidence could be further rendered to fill these gaps to improve the applicability.

We found that few TMFs concerning “processes” targeted the scale-up phase, which is consistent with a previous review [11]. This could be because implementation projects were usually conducted in a single-site setting. With the development of this field, large-scale projects like multi-region programs are expected to grow, which could trigger the development of TMFs focusing on scale-up. Also, attention should be paid to the stage sequence, as many TMFs mention the nonlinear relationships among stages. However, TMF developers seldom tested this assumption, which made it challenging during application as TMF users may not be able to decide when and how to move back and forth during the process [8].

Among TMFs covering the theme of “outcomes,” few of them examined the interactions among different outcomes. However, identifying the relationship between outcomes is important when choosing target implementation outcomes for evaluation studies. Research has indicated that outcomes like appropriateness, feasibility, and cost may serve as the prerequisite for adoption, and outcomes like penetration may have a relation to sustainability [130]. Therefore, future studies are needed to comprehensively understand the relationship between different outcomes.

Only some authors put forward a relevant research method for their TMFs. Applying appropriate research methods is imperative to prevent the misuse or superficial use of conceptual frameworks, especially for novices. Recommendations of proper research methods for the TMFs may facilitate the standardization of the application process, which would contribute to the comparison and synthesis of empirical evidence and refinement of the current TMFs. In addition, few TMFs that provided measurable scales were tested, making their sensitivity and specificity unclear when assessing the objectives.

In terms of the analysis level, we found that “team” was one of the least studied levels. This finding aligned with a scoping review suggesting that team-level determinants were almost entirely overlooked in the context classification [131]. Without proper position and definition, this analysis level lacked attention. However, according to the same scoping review [131], the team was a vital component of an organization and contributed to the integration of care provision. Thus, future research is required to emphasize team or group-level analyses. The policy level was another area that was not as well studied, which is consistent with the findings of previous reviews [7, 12]. A potential explanation might be the independent development of implementation science and policy implementation research, which means that implementation in healthcare is not often studied using a policy lens or a societal perspective [131]. However, evidence indicated that policymaking explicitly facilitated the adoption and implementation of evidence-based practices [132, 133]. Thus, knowledge exchange between these two separate fields would be beneficial for a more comprehensive understanding of the challenges of implementation.

Generalizable TMFs have benefits in their flexibility to apply the TMF. However, specificity is required when a specific population or setting is studied. Influential factors like culture, religious traditions, and political backgrounds may be important in determining their successful implementation. Even though developing new TMFs are not encouraged when a proliferation of TMFs has already been available, researchers have suggested taking the context, such as settings and target audience, into account when making decisions on adapting an existing TMF or developing a new one.

Regarding testability, only some TMF developers explicitly articulate the hypothesis, assumption, or propositions. Lacking an explicit hypothesis before TMF formulation indicates that the primary function of the TMF may be the interpretation of a phenomenon instead of theory verification. The complexity of the implementation process may also make the explicit hypothesis too intricate to formulate. Nevertheless, diagnosis or prediction TMFs could help researchers to generate hypotheses with the indicated relationships between elements. Besides, empirical support could be in a significant amount of TMFs. The main empirical evidence utilized was either case studies or literature reviews. Case studies were often used retrospectively to testify the TMFs, while literature reviews were employed prospectively to generate the evidence base for the theoretical models. However, we found that empirical evidence was often missing to support the relationships claimed by the developers. This finding is consistent with Albers et al., who reported that the clarity of how the individual elements of the TMFs worked together was limited by evidence-based support [8].

Regarding quantification, we added up the scores across the ten-measurement metrics for each TMF and found that only one-third met our priori standard of a high-quality framework, even though most of the TMFs had a score between 5 and 7. This might indicate that theory quantity is given more attention over quality at the beginning stage of a new field to build a comprehensive system. However, with the proliferation of TMFs identified by our study and other reviews, we suggest focusing on the quality of the theoretical framework rather than the quantity. The vertical analysis also sheds light on the potential challenges of the newly developed discipline, which is the rigorousness of scientific research. The domain of testability got the lowest score, and few TMFs were designed with a clear hypothesis, assumption, or proposition. Future studies should pay more attention to hypothesis clarification when developing frameworks.

### Strengths

This study further contributed to the literature by reporting the quality of existing TMFs in terms of their usability, applicability, and testability. To our knowledge, this is the first scoping review to conduct a quality assessment for implementation science TMFs. By following the structured appraisal scale [4], we were able to comprehensively understand current conceptual development. We were also able to go beyond the descriptive summarization, which was the main method employed by previous reviews [7, 10–13], to describe the basic features of TMFs.

### Limitations

There are several limitations with this study. First, the adapted appraisal framework was primarily designed for comparing TMFs using a Likert scale ranging from 0 to 2 [4]. To accommodate the primary purpose of this review, which was to appraise the quality and understand the development of TMFs instead of the individual frameworks, and to simplify the analysis, we adjusted the scoring criteria to a yes or no scheme. This dichotomy method may hinder the depth of the analysis of one criterion and thus may lack comprehensiveness. However, this would not affect the implications for the whole body of the TMFs.

Second, this review adopted multiple classification schemes (e.g., context levels, process stages, and strategy types). These classifications were not identical in different articles, and there are various ways to make categorizations. Thus, confusion may occur. Future research on standardized taxonomies of certain concepts is needed to facilitate shared understanding and communication.

Third, data collection was performed by a single reviewer (Y. X. W.), with assistance from several professionals (Professor Nilsen Per and Professor Eliza Wong) in this field. The single reviewer may cause bias in the decisions on article inclusion. However, clear definitions of the inclusion criteria and professional confirmations were likely to minimize the uncertainties.

## Conclusions

By analyzing implementation science TMFs on their usability, applicability, and testability, this review found that common limitations existed among the current TMFs. For usability, the causal relationship can be further clarified and verified among elements of the TMFs. Meanwhile, researchers can provide more practical application guidance. Regarding the applicability, more attention could be paid to the coverage of implementation science constructs, the depth of understanding of each construct, and the appropriate research methods and measurement metrics recommendations. Concerning testability, hypothesis, assumptions, and propositions could be more explicitly stated during the development of TMFs. The findings of this review provide insights for TMF developers for future theoretical advancement. Researchers are encouraged to apply TMFs in all implementation science studies [11], by which reciprocal benefits could occur in discipline developments and health service improvements.

### Supplementary Information

**Additional file 1.**  Data extraction items and definitions.

**Additional file 2.** Purpose and Characteristics of 143 Theories, Models, and Frameworks (TMFs).

**Additional file 3.** Preferred Reporting Items for Systematic reviews and Meta-Analyses extension for Scoping Reviews (PRISMA-ScR) Checklist.

## Data Availability

Not applicable.

## Abbreviations

TMFs: Theories, models, and frameworks
EBPs: Evidence-based practices
T-CaST: Theory, Model, and Framework Comparison and Selection Tool
CFIR: Consolidated Framework of Implementation Research
ERIC: Expert Recommendations for Implementing Change
EPOC: Cochrane Effective Practice and Organization of Care
