# Supplementary material for: A scoping review of implementation science theories, models, and frameworks — an appraisal of purpose, characteristics, usability, applicability, and testability
Source: Implement Sci. 2023 Sep 19;18:43. doi: 10.1186/s13012-023-01296-x (PMC10507824; doi:10.1186/s13012-023-01296-x)
Supplement: Supplementary file 1 — Additional file 1. Data extraction items and definitions. [file 13012_2023_1296_MOESM1_ESM.docx]

**Additional File 1: Data extraction items and definitions**

| PURPOSE |  |
| --- | --- |
| 1. Identify barriers and facilitators | |
| 1. Inform data collection | |
| 1. Guide implementation planning | |
| 1. Enhance conceptual clarity | |
| 1. Specify process of implementation | |
| 1. Frame evaluation | |
| 1. Inform data analysis | |
| 1. Guide design or selection of IS Strategies | |
| 1. Specify relationship between constructs | |
| CHARACTERISTICS OF THEORIES, MODELS AND FRAMEWORKS (TMFs) | |
| 1. TMFs category |  |
| 1. Determinant frameworks | Classes or domains of determinants that are hypothesized or have been found to influence implementation outcomes |
| 1. Evaluation frameworks | Provide a structure for evaluating implementation endeavors |
| 1. Strategy frameworks | Provide a structure of implementation interventions to facilitate the implementation process |
| 1. Measurement frameworks | Provide structured measurement metrics to implementation constructs or influential factors |
| 1. Process models | Specify steps in the process of translating research into practice. |
| 1. Theoretical underpinning | Whether frameworks/models were built on grand theories or exited conceptual frameworks |
| 1. Theory level | The degree to which their generalization are ordered, and the level of abstraction at which they explore social phenomena |
| 1. Level of analysis |  |
| 1. Descriptive | Describe the properties, characteristics, and/or qualities of implementation. |
| 1. Diagnostic | Specify linkage and/or relationships between framework concepts. |
| 1. Predictive | Provide hypothesis or propose directional relationships between concepts of implementation. |
| 1. Prescriptive | Provide directions on the implementation process via a series of steps or procedures |
| TMFs APPRAISAL CRITERIA | |
| 1. Usability |  |
| 1. Relevancy of constructs | TMFs articulated the relevancy of constructs |
| 1. Diagram of TMFs | Have a clear and useful figure depicting included constructs and relationships among them |
| 1. Guidance for application | Provision of a step to step approach for applying it |
| 1. Change strategies | TMF Provides methods for promoting implementation in practice |
| 1. Mechanism/relationships between constructs | TMF provides an explanation of how included constructs influence implementation or each other, |
| 1. Applicability |  |
| 1. Implementation science constructs | TMFs includes a relevant implementation science constructs |
| 1. Context | Variables that may affect the implementation process and outcome. Also termed facilitators and barriers or determinants of practice. |
| 1. Strategy | Targeted efforts (method, technique or activity) designed to enhance moving of an innovation into use and integrating into routine practice. |
| 1. Outcome | Metrics to measure the successful implementation of new treatments, programs and services. |
| 1. Stages of implementation process | The breakdown of the complete implementation process |
| 1. Fidelity/adaptation | Fidelity is the degree to which interventions are implemented as intended by those who developed or designed the intervention. Adaptation is defined as a change to the content or delivery of an EBP that is designed to tailor the EBP to the needs of a given context. |
| 1. Sustainability | In healthcare, it often refers to continuation of the health benefits, continuation of initiative activities, continuation of capacity built into the workforce and continued financial viability. |
| 1. Proposed research/measurement methods | A particular method of research(e.g., interview, surveys, focus group, chart review) or measurement metrics can be used with TMFs |
| 1. Level of change | TMFs address a relevant analytic level(e.g., individual, organizational, community) |
| 1. Generalizability | TMFs are generalizable to other disciplines(e.g., education, health service, social work), settings(e.g., schools, hospitals, community-based organizations) and populations |
| 1. Setting | Physical environment where intervention takes place |
| 1. Target audience | Population received the intervention |
| 1. Innovation type |  |
| 1. Intervention | Including interventions, programs, innovations, complex innovations, shared-decision making, technologies, evidence-based practices, telehealth, service, QI project, integrated care |
| 1. Guideline | Including clinical practice, best practice, guideline, depresribing, process |
| 1. Knowledge | Including knowledge, research, ethical norms |
| 1. Policy | - |
| 1. Implementation programs | - |
| 1. Testability |  |
| 1. Explicit hypothesis | TMFs propose testable hypotheses |
| 1. Evidence of change mechanism | TMFs include meaningful, face-valid evidence of proposed relationship |
| 1. Empirical support | TMFs contribute to evidence base and TMF development because it has been used in empirical studies |

1. **Quantification analysis**

| Items included in quantification analysis | | | |
| --- | --- | --- | --- |
| 1. Relevancy of constructs | | | |
| 1. Diagram of TMFs | | | |
| 1. Guidance for application | |  | |
| 1. Change strategies |  | | |
| 1. Mechanism/relationships between constructs | | |  |
| 1. Proposed research/measurement methods | | | |
| 1. Generalizability | | | |
| 1. Explicit hypothesis | | | |
| 1. Evidence of change mechanism | | | |
| 1. Empirical support | | | |
