# Supplementary material for: A scoping review of implementation science theories, models, and frameworks — an appraisal of purpose, characteristics, usability, applicability, and testability
Source: Implement Sci. 2023 Sep 19;18:43. doi: 10.1186/s13012-023-01296-x (PMC10507824; doi:10.1186/s13012-023-01296-x)
Supplement: Supplementary file 2 — Additional file 2. Purpose and Characteristics of 143 Theories, Models, and Frameworks (TMFs). [file 13012_2023_1296_MOESM2_ESM.docx]

**Additional file 2. Purpose and Characteristics of 143 Theories, Models, and Frameworks (TMFs)**

| **Reference** | **Name of the TMFs** | **Purpose** | **Characteristics of TMFs** | | | | | |
| --- | --- | --- | --- | --- | --- | --- | --- | --- |
|  |  |  | **TMFs category** | | **Theory underpinned** | **Established conceptual framework underpinned** | **TMFs theory level** | **TMFs level of analytics** |
| (1) | Theoretical domains framework | Identify barriers and facilitators | Determinant framework |  | Y | Y | Mid-range theory | Descriptive |
| (2) | CASCADA | Identify barriers and facilitators | Determinant framework |  | Y | Y | Mid-range theory | Predictive |
| (3) | Stakeholder analysis | Inform data analysis | Determinant framework | Measurement framework | Y | N | Mid-range theory | Descriptive |
| (4) | Framework by Dy et al. | Identify barriers and facilitators | Determinant framework |  | N | Y | Program theory | Diagnostic |
| (5) | Framework by Gagliardi et al. | Identify barriers and facilitators | Determinant framework |  | N | N | Mid-range theory | Descriptive |
| (6) | Framework by Roger et al. | Identify barriers and facilitators | Determinant framework |  | N | Y | Mid-range theory | Diagnostic |
| (7, 8) | Complex Adaptive Science | Enhance conceptual clarity | Determinant framework |  | Y | N | Mid-range theory | Predictive |
| (9) | CFIR | Identify barriers and facilitators | Determinant framework |  | N | Y | Mid-range theory | Descriptive |
| (10) | CEDI Framework | Identify barriers and facilitators | Determinant framework |  | Y | Y | Mid-range theory | Descriptive |
| (11) | Complex Theory Core Concepts Framework | Identify barriers and facilitators | Determinant framework |  | Y | N | Mid-range theory | Diagnostic |
| (12) | EPIS | Identify barriers and facilitators | Determinant framework | Process model | N | Y | Mid-range theory | Diagnostic |
| (13) | eUTC | Identify barriers and facilitators | Determinant framework |  | N | Y | Mid-range theory | Prescriptive |
| (14) | General theory of Implementation (Extended NPT) | Enhance conceptual clarity | Determinant framework |  | Y | Y | Mid-range theory | Diagnostic |
| (15) | TICD Checklist | Identify barriers and facilitators | Determinant framework |  | N | N | NA | Descriptive |
| (16) | IPAT | Identify barriers and facilitators | Determinant framework | Measurement framework | N | Y | NA | Predictive |
| (17) | IMHI | Identify barriers and facilitators | Determinant framework |  | Y | N | Mid-range theory | Predictive |
| (18) | ISF | Identify barriers and facilitators | Determinant framework |  | N | Y | Mid-range theory | Descriptive |
| (19) | Model of alignment | Identify barriers and facilitators | Determinant framework |  | N | N | Mid-range theory | Descriptive |
| (20) | NASSS (Influence on the adoption, nonadoption, abandonment, spread, scale-up, and sustainability) | Identify barriers and facilitators | Determinant framework | Evaluation framework | Y | N | Mid-range theory | Diagnostic |
| (21) | NPT | Identify barriers and facilitators | Determinant framework | Evaluation framework | Y | N | Mid-range theory | Diagnostic |
| (22) | Nurse LEAD-IT-mHealth | Identify barriers and facilitators | Determinant framework |  | N | Y | Mid-range theory | Predictive |
| (23) | Implementation climate theory | Identify barriers and facilitators | Determinant framework |  | Y | N | Mid-range theory | Diagnostic |
| (24) | i-PARiHS | Guide implementation planning | Determinant framework |  | N | Y | Mid-range theory | Diagnostic |
| (25) | Performance Improvement Model | Specify relationship between constructs | Determinant framework |  | Y | N | Mid-range theory | Predictive |
| (26) | Process redesign(PR) framework | Identify barriers and facilitators | Determinant framework |  | N | Y | Mid-range theory | Descriptive |
| (27) | Integrated Model of Program Implementation | Identify barriers and facilitators | Determinant framework |  | N | Y | Mid-range theory | Diagnostic |
| (28) | Anderson-PARiHS hybrid framework | Identify barriers and facilitators | Determinant framework |  | N | Y | Program theory | Descriptive |
| (29) | ITIM | Identify barriers and facilitators | Determinant framework |  | N | Y | Mid-range theory | Descriptive |
| (30) | Framework by Shediac-Rizkalla & Bone | Identify barriers and facilitators | Determinant framework |  | N | N | Program theory | Diagnostic |
| (31) | Social ecological model | Identify barriers and facilitators | Determinant framework |  | Y | N | Classic theory | Descriptive |
| (32) | Social network theory | Identify barriers and facilitators | Determinant framework |  | Y | N | Classic theory | Diagnostic |
| (33) | Conceptual framework of supporting factors | Identify barriers and facilitators | Determinant framework |  | N | N | Mid-range theory | Descriptive |
| (34) | Multilevel context of cancer care | Identify barriers and facilitators | Determinant framework |  | N | Y | Program theory | Descriptive |
| (35) | UTAUT | Identify barriers and facilitators | Determinant framework | Measurement framework | N | Y | Mid-range theory | Diagnostic |
| (36) | Contextual factor framework | Identify barriers and facilitators | Determinant framework |  | N | Y | Mid-range theory | Descriptive |
| (37) | Expand CFIR | Identify barriers and facilitators | Determinant framework |  | Y | Y | Mid-range theory | Descriptive |
| (38) | RTI-model | Identify barriers and facilitators | Determinant framework |  | Y | Y | Program theory | Diagnostic |
| (39) | Health equity implementation framework | Identify barriers and facilitators | Determinant framework |  | N | Y | Mid-range theory | Descriptive |
| (40) | Clinical adoption framework | Identify barriers and facilitators | Determinant framework |  | Y | Y | Mid-range theory | Descriptive |
| (41) | COACH | Identify barriers and facilitators | Determinant framework |  | N | Y | NA | Descriptive |
| (42) | Determinants of Implementation Effectiveness | Identify barriers and facilitators | Determinant framework |  | N | Y | Mid-range theory | Descriptive |
| (43) | PRISM(A Practical, Robust Implementation and Sustainability Model) | Identify barriers and facilitators | Determinant framework | Evaluation framework | Y | Y | Mid-range theory | Prescriptive |
| (44) | CICI | Identify barriers and facilitators | Determinant framework |  | Y | Y | Mid-range theory | Descriptive |
| (45) | TAM | Identify barriers and facilitators | Determinant framework |  | Y | N | Classic theory | Predictive |
| (46) | AIF | Guide implementation planning | Determinant framework | Process model | N | N | Mid-range theory | Descriptive |
| (47) | AACTT(action, actor, context, target, time) | Identify barriers and facilitators | Determinant framework |  | N | Y | Mid-range theory | Descriptive |
| (48) | Organizational readiness for change | Enhance conceptual clarity | Determinant framework | Strategy framework | Y | N | Mid-range theory | Predictive |
| (49) | Social Interface Model | Identify barriers and facilitators | Determinant framework |  | Y | N | Mid-range theory | Predictive |
| (50) | Framework by Grol | Identify barriers and facilitators | Determinant framework |  | N | N | Mid-range theory | Descriptive |
| (51) | Conceptual framework of integration intervention into health system | Identify barriers and facilitators | Determinant framework |  | Y | N | Mid-range theory | Diagnostic |
| (52) | Tool+ team+ routine | Guide design or selection of IS Strategies | Determinant framework |  | Y | Y | Mid-range theory | Diagnostic |
| (53) | PNE framework | Identify barriers and facilitators | Determinant framework |  | N | Y | Mid-range theory | Diagnostic |
| (54) | Dynamic sustainability framework | Enhance conceptual clarity | Determinant framework |  | N | N | NA | Diagnostic |
| (55) | Deprescribing framework | Frame evaluation | Determinant framework | Evaluation framework | N | Y | Program theory | Descriptive |
| (56) | Framework by Cochrane et al. | Identify barriers and facilitators | Determinant framework |  | N | N | Mid-range theory | Descriptive |
| (57) | Framework by Gurses et al. | Identify barriers and facilitators | Determinant framework |  | N | N | Mid-range theory | Diagnostic |
| (58) | Ecological framework | Identify barriers and facilitators | Determinant framework |  | N | Y | Mid-range theory | Predictive |
| (59) | Understanding user-context | Identify barriers and facilitators | Determinant framework |  | N | N | Mid-range theory | Diagnostic |
| (60) | model by Landry et al. | Identify barriers and facilitators | Determinant framework |  | Y | N | Mid-range theory | Diagnostic |
| (61) | Model by Ferile and Shortell | Identify barriers and facilitators | Determinant framework |  | N | N | NA | Descriptive |
| (62) | Framework by Zhang et al. | Identify barriers and facilitators | Determinant framework |  | N | Y | Mid-range theory | Descriptive |
| (63) | Framework by Chen et al. | Identify barriers and facilitators | Determinant framework |  | N | Y | Mid-range theory | Descriptive |
| (64) | Model for evaluation of implementation program and professional pharmacy service | Frame evaluation | Evaluation framework |  | N | Y | Mid-range theory | Prescriptive |
| (65) | Framework and coding system for modification and adaption of EBI | Inform data collection | Evaluation framework |  | N | N | Mid-range theory | Descriptive |
| (66) | FRAME | Inform data collection | Evaluation framework |  | N | Y | Mid-range theory | Descriptive |
| (67) | RE-AIM | Frame evaluation | Evaluation framework |  | Y | N | Mid-range theory | Descriptive |
| (68) | FORECAST 2.0 | Frame evaluation | Evaluation framework |  | N | Y | Mid-range theory | Prescriptive |
| (69) | Process evaluation model | Frame evaluation | Evaluation framework | Process model | N | N | Program theory | Descriptive |
| (70) | HOT-fit | Frame evaluation | Evaluation framework |  | N | Y | Mid-range theory | Diagnostic |
| (71) | Conceptual framework for implementation outcomes | Frame evaluation | Evaluation framework |  | Y | Y | Mid-range theory | Descriptive |
| (72) | QUERI Impact Framework | Frame evaluation | Evaluation framework |  | N | Y | Program theory | Descriptive |
| (73) | Science Impact Framework | Frame evaluation | Evaluation framework |  | N | Y | Program theory | Descriptive |
| (74) | Gavi Full Country Evaluation Framework | Frame evaluation | Evaluation framework |  | Y | N | Program theory | Descriptive |
| (75) | The Stages of implementation completion(SIC) | Frame evaluation | Evaluation framework |  | N | N | NA | Diagnostic |
| (76) | Conceptual framework for implementation fidelity | Enhance conceptual clarity | Evaluation framework |  | N | N | Mid-range theory | Diagnostic |
| (77) | Framework of fidelity of intervention and implementation | Frame evaluation | Evaluation framework |  | N | Y | Mid-range theory | Diagnostic |
| (78) | The Value Equation | Specify relationship between constructs | Evaluation framework |  | Y | N | Mid-range theory | Predictive |
| (79) | MADI | Frame evaluation | Evaluation framework |  | N | Y | Mid-range theory | Diagnostic |
| (80) | Reflective framework for successful IS implementation | Guide implementation planning | Evaluation framework |  | Y | Y | Mid-range theory | Descriptive |
| (81) | LADDERS | Frame evaluation | Evaluation framework |  | N | N | Mid-range theory | Diagnostic |
| (82) | Consolidated framework for sustainability constructs in healthcare | Enhance conceptual clarity | Evaluation framework |  | N | N | Mid-range theory | Descriptive |
| (83) | Recommendation for designing a sustainability evaluation | Frame evaluation | Evaluation framework | Measurement framework | N | N | Program theory | Descriptive |
| (84) | TyDI | Frame evaluation | Evaluation framework |  | N | N | NA | Prescriptive |
| (85) | Behavioural change wheel | Guide design or selection of IS Strategies | Strategy framework |  | Y | N | Mid-range theory | Diagnostic |
| (86) | Policy Ecology Framework | Guide design or selection of IS Strategies | Strategy framework |  | N | N | NA | Descriptive |
| (87) | Structural competency framework | Guide design or selection of IS Strategies | Strategy framework |  | N | N | Mid-range theory | Descriptive |
| (88) | ARC | Guide design or selection of IS Strategies | Strategy framework | Process model | Y | Y | Mid-range theory | Descriptive |
| (89) | He Pikinga Waiora | Guide implementation planning | Strategy framework |  | Y | N | Mid-range theory | Descriptive |
| (90) | DART | Guide design or selection of IS Strategies | Strategy framework | Determinant framework | N | N | NA | Descriptive |
| (91) | EBSIS | Guide design or selection of IS Strategies | Strategy framework |  | N | Y | Mid-range theory | Descriptive |
| (92) | INSPIRE | Guide design or selection of IS Strategies | Strategy framework |  | Y | Y | Program theory | Prescriptive |
| (93) | Taxonomy of methods for implementation change | Guide design or selection of IS Strategies | Strategy framework |  | Y | N | Mid-range theory | Descriptive |
| (94) | Multi-stakeholder partnerships | Enhance conceptual clarity | Strategy framework |  | Y | N | Mid-range theory | Descriptive |
| (95) | Community-integrated intermediary | Enhance conceptual clarity | Strategy framework |  | Y | N | Mid-range theory | Descriptive |
| (96) | Model of organizational integration, implementation effort and performance | Enhance conceptual clarity | Strategy framework |  | Y | N | Mid-range theory | Predictive |
| (97) | Conceptual framework for organizational learning | Enhance conceptual clarity | Strategy framework |  | Y | N | Mid-range theory | Diagnostic |
| (98, 99) | ERIC | Guide design or selection of IS Strategies | Strategy framework |  | N | Y | Mid-range theory | Descriptive |
| (100) | Quality Implementation Tool | Guide implementation planning | Strategy framework |  | N | Y | NA | Prescriptive |
| (101) | Practice change development model | Guide design or selection of IS Strategies | Strategy framework |  | Y | Y | Mid-range theory | Predictive |
| (102) | System change framework | Specify relationship between constructs | Strategy framework |  | Y | N | Mid-range theory | Diagnostic |
| (103) | Strategic action field framework | Specify relationship between constructs | Strategy framework |  | Y | N | Mid-range theory | Diagnostic |
| (104) | Implementation mapping | Guide design or selection of IS Strategies | Strategy framework |  | N | Y | NA | Prescriptive |
| (105) | Conceptual framework of research to evidence-informed decision making | Guide design or selection of IS Strategies | Strategy framework |  | N | Y | Mid-range theory | Prescriptive |
| (106) | Implementation research logic model | Specify relationship between constructs | Strategy framework |  | N | Y | Mid-range theory | Descriptive |
| (107) | Information technology implementation framework | Guide design or selection of IS Strategies | Strategy framework | Evaluation framework | Y | Y | Mid-range theory | Prescriptive |
| (108) | Framework for enhancing the value of D&I research (reporting guideline) | Guide design or selection of IS Strategies | Strategy framework |  | N | N | NA | Descriptive |
| (109) | Empowerment implementation | Guide design or selection of IS Strategies | Strategy framework |  | Y | N | Mid-range theory | Prescriptive |
| (110) | Conceptual framework for implementation research | Enhance conceptual clarity | Strategy framework |  | Y | Y | Mid-range theory | Descriptive |
| (111) | AIDED | Guide implementation planning | Strategy framework | Process model | N | N | NA | Prescriptive |
| (112) | AIMD | Guide design or selection of IS Strategies | Strategy framework |  | N | Y | Mid-range theory | Descriptive |
| (113) | AICP | Guide implementation planning | Strategy framework |  | N | Y | Mid-range theory | Descriptive |
| (114) | Organizational readiness for implementing change | Inform data analysis | Measurement framework |  | Y | N | NA | Descriptive |
| (115) | CWDIF | Specify process of implementation | Process model |  | N | Y | Program theory | Prescriptive |
| (116) | QIF(Quality implementation framework) | Guide implementation planning | Process model |  | N | Y | Mid-range theory | Prescriptive |
| (117) | FISpH | Specify process of implementation | Process model |  | N | Y | Program theory | Prescriptive |
| (118) | Iowa Model | Specify process of implementation | Process model |  | N | Y | Mid-range theory | Prescriptive |
| (119) | KTA | Specify process of implementation | Process model |  | Y | N | Mid-range theory | Prescriptive |
| (120) | Stage-based framework for implementation of early childhood program | Guide implementation planning | Process model |  | N | Y | Program theory | Prescriptive |
| (121) | Ottawa model of research use(OMRU) | Specify process of implementation | Process model |  | N | Y | Mid-range theory | Prescriptive |
| (122) | ADAPT-ITT Model | Guide implementation planning | Process model |  | N | N | NA | Prescriptive |
| (123) | Dynamic adaptation process(DAP) | Guide implementation planning | Process model |  | N | Y | Mid-range theory | Prescriptive |
| (124) | IDEA | Guide implementation planning | Process model |  | N | N | NA | Prescriptive |
| (125) | EquLR | Guide implementation planning | Process model |  | N | Y | Mid-range theory | Prescriptive |
| (126) | Comprehensive framework for nutrition implementation science | Specify process of implementation | Process model |  | Y | N | Mid-range theory | Prescriptive |
| (127) | CITRA Research-Practice Consensus-Workshop Model | Specify process of implementation | Process model |  | N | Y | Program theory | Prescriptive |
| (128) | Implementation of Change Model | Guide implementation planning | Process model |  | Y | unknown | Mid-range theory | Prescriptive |
| (129) | ADAPTS | Specify process of implementation | Process model |  | Y | Y | Mid-range theory | Prescriptive |
| (130) | MRC(Medical Research Council's evaluation framework) | Frame evaluation | Process model | Evaluation framework | N | Y | Mid-range theory | Prescriptive |
| (131) | Design-focused implementation | Specify process of implementation | Process model |  | N | Y | Program theory | Prescriptive |
| (132) | Research to practice framework for technology transfer | Specify process of implementation | Process model |  | N | N | NA | Prescriptive |
| (133) | Stetler’s Model | Specify process of implementation | Process model |  | N | Y | Mid-range theory | Prescriptive |
| (134) | Guide on spread and sustainability | Guide implementation planning | Process model |  | N | Y | Mid-range theory | Prescriptive |
| (135) | Moving from ought to is | Specify process of implementation | Process model |  | N | Y | Mid-range theory | Prescriptive |
| (136) | Framework for Going to Full Scale | Specify process of implementation | Process model |  | N | Y | Mid-range theory | Prescriptive |
| (137) | CTAP | Specify process of implementation | Process model |  | N | Y | Mid-range theory | Prescriptive |
| (138) | Transcreational framework | Guide implementation planning | Process model |  | Y | Y | Mid-range theory | Prescriptive |
| (139) | RBM(result based management) | Guide implementation planning | Process model |  | N | N | NA | Descriptive |
| (140) | K2A | Specify process of implementation | Process model |  | N | Y | Mid-range theory | Prescriptive |
| (141) | ACE Star Model of knowledge transformation | Specify process of implementation | Process model |  | N | N | NA | Prescriptive |
| (142) | Model by Majdzadeh et al. | Guide design or selection of IS Strategies | Process model | Determinant framework | N | N | Mid-range theory | Prescriptive |
| (143) | Model by Davis et al. | Specify process of implementation | Process model |  | N | Y | Mid-range theory | Prescriptive |
| (144) | Model by Pronovost et al. | Specify process of implementation | Process model |  | N | N | NA | Prescriptive |
| (145) | Pathway for evidence-based nursing practice | Specify process of implementation | Process model |  | N | Y | Mid-range theory | Prescriptive |

1. Cane J, O'Connor D, Michie S. Validation of the theoretical domains framework for use in behaviour change and implementation research. Implementation Science. 2012;7.

2. Andersson N, Beauchamp M, Nava-Aguilera E, Paredes-Solis S, Sajna M. The women made it work: fuzzy transitive closure of the results chain in a dengue prevention trial in Mexico. BMC public health. 2017;17.

3. Balane MA, Palafox B, Palileo-Villanueva LM, McKee M, Balabanova D. Enhancing the use of stakeholder analysis for policy implementation research: towards a novel framing and operationalised measures. BMJ global health. 2020;5(11).

4. Dy SM, Ashok M, Wines RC, Smith LR. A Framework to Guide Implementation Research for Care Transitions Interventions. J Healthc Qual. 2015;37(1):41-54.

5. Gagliardi AR, Brouwers MC, Palda VA, Lemieux-Charles L, Grimshaw JM. How can we improve guideline use? A conceptual framework of implementability. Implementation Science. 2011;6.

6. Rogers L, De Brun A, McAuliffe E. Development of an integrative coding framework for evaluating context within implementation science. BMC medical research methodology. 2020;20(1).

7. Plsek PE, Greenhalgh T. Complexity science - The challenge of complexity in health care. Brit Med J. 2001;323(7313):625-8.

8. Plsek PE, Wilson T. Complexity science - Complexity, leadership, and management in healthcare organisations. Bmj-British Medical Journal. 2001;323(7315):746-9.

9. Damschroder LJ, Aron DC, Keith RE, Kirsh SR, Alexander JA, Lowery JC. Fostering implementation of health services research findings into practice: a consolidated framework for advancing implementation science. Implementation Science. 2009;4.

10. Shea CM, Young TL, Powell BJ, Rohweder C, Enga ZK, Scott JE, et al. Researcher readiness for participating in community-engaged dissemination and implementation research: a conceptual framework of core competencies. Translational behavioral medicine. 2017;7(3):393-404.

11. Chandler J, Rycroft-Malone J, Hawkes C, Noyes J. Application of simplified Complexity Theory concepts for healthcare social systems to explain the implementation of evidence into practice. J Adv Nurs. 2016;72(2):461-80.

12. Aarons GA, Hurlburt M, Horwitz SM. Advancing a Conceptual Model of Evidence-Based Practice Implementation in Public Service Sectors. Administration and Policy in Mental Health and Mental Health Services Research. 2011;38(1):4-23.

13. Kayser L, Kushniruk A, Osborne RH, Norgaard O, Turner P. Enhancing the Effectiveness of Consumer-Focused Health Information Technology Systems Through eHealth Literacy: A Framework for Understanding Users' Needs. JMIR human factors. 2015;2(1):e9.

14. May C. Towards a general theory of implementation. Implementation Science. 2013;8.

15. Flottorp SA, Oxman AD, Krause J, Musila NR, Wensing M, Godycki-Cwirko M, et al. A checklist for identifying determinants of practice: A systematic review and synthesis of frameworks and taxonomies of factors that prevent or enable improvements in healthcare professional practice. Implementation Science. 2013;8.

16. Hartveit M, Hovlid E, Nordin MHA, Ovretveit J, Bond GR, Biringer E, et al. Measuring implementation: development of the implementation process assessment tool (IPAT). BMC health services research. 2019;19(1).

17. Connor YO, Donoghue JO, Reilly PO. Infusion of Mobile Health Systems in the NHS: An Empirical Study. Proceedings of the 6th European Conference on Information Management and Evaluation. 2012:226-33.

18. Wandersman A, Duffy J, Flaspohler P, Noonan R, Lubell K, Stillman L, et al. Bridging the gap between prevention research and practice: The interactive systems framework for dissemination and implementation. American Journal of Community Psychology. 2008;41(3-4):171-81.

19. Pannick S, Sevdalis N, Athanasiou T. Beyond clinical engagement: a pragmatic model for quality improvement interventions, aligning clinical and managerial priorities. BMJ Qual Saf. 2016;25(9):716-25.

20. Greenhalgh T, Wherton J, Papoutsi C, Lynch J, Hughes G, A'Court C, et al. Beyond Adoption: A New Framework for Theorizing and Evaluating Nonadoption, Abandonment, and Challenges to the Scale-Up, Spread, and Sustainability of Health and Care Technologies. Journal of Medical Internet Research. 2017;19(11).

21. May C, Finch T. Implementing, Embedding, and Integrating Practices: An Outline of Normalization Process Theory. Sociology. 2009;43(3):535-54.

22. Ronquillo C, Dahinten VS, Bungay V, Currie LM. The Nurse LEADership for Implementing Technologies - Mobile Health Model (Nurse LEAD-IT - mHealth). Nursing leadership (Toronto, Ont). 2019;32(2):71-84.

23. Klein KJ, Sorra JS. The challenge of innovation implementation. Acad Manage Rev. 1996;21(4):1055-80.

24. Harvey G, Kitson A. PARIHS revisited: from heuristic to integrated framework for the successful implementation of knowledge into practice. Implementation Science. 2016;11.

25. Hambrick DC, Mason PA. Upper Echelons - the Organization as a Reflection of Its Top Managers. Acad Manage Rev. 1984;9(2):193-206.

26. Ashok M, Hung D, Rojas-Smith L, Halpern MT, Harrison M. Framework for Research on Implementation of Process Redesigns. Qual Manag Health Care. 2018;27(1):17-23.

27. Berkel C, Mauricio AM, Schoenfelder E, Sandler IN. Putting the Pieces Together: An Integrated Model of Program Implementation. Prev Sci. 2011;12(1):23-33.

28. Rongey C, Asch S, Knight SJ. Access to care for vulnerable veterans with hepatitis C: a hybrid conceptual framework and a case study to guide translation. Translational behavioral medicine. 2011;1(4):644-51.

29. Schoville RR, Titler MG. Guiding Healthcare Technology Implementation A New Integrated Technology Implementation Model. Cin-Computers Informatics Nursing. 2015;33(3):99-107.

30. Mayhew SH, Sweeney S, Warren CE, Collumbien M, Ndwiga C, Mutemwa R, et al. Numbers, systems, people: how interactions influence integration. Insights from case studies of HIV and reproductive health services delivery in Kenya. Health policy and planning. 2017;32:67-81.

31. Stokols D, Allen J, Bellingham RL. The social ecology of health promotion: Implications for research and practice. American Journal of Health Promotion. 1996;10(4):247-51.

32. Lin CA. Network models of the diffusion of innovations - Valente,TW. Journalism Mass Comm. 1996;73(4):1008-9.

33. Hodge LM, Turner KMT. Sustained Implementation of Evidence-based Programs in Disadvantaged Communities: A Conceptual Framework of Supporting Factors. American Journal of Community Psychology. 2016;58(1-2):192-210.

34. Taplin SH, Yabroff KR, Zapka J. A Multilevel Research Perspective on Cancer Care Delivery: The Example of Follow-Up to An Abnormal Mammogram. Cancer Epidemiology Biomarkers & Prevention. 2012;21(10):1709-15.

35. Venkatesh V, Morris MG, Davis GB, Davis FD. User acceptance of information technology: Toward a unified view. Mis Quart. 2003;27(3):425-78.

36. Vanderkruik R, McPherson ME. A Contextual Factors Framework to Inform Implementation and Evaluation of Public Health Initiatives. American Journal of Evaluation. 2017;38(3):348-59.

37. Senier L, McBride CM, Ramsey AT, Bonham VL, Chambers DA. Blending Insights from Implementation Science and the Social Sciences to Mitigate Inequities in Screening for Hereditary Cancer Syndromes. International journal of environmental research and public health. 2019;16(20).

38. de Wit M, Kleijnen M, Lissenberg-Witte B, van Uden-Kraan C, Millet K, Frambach R, et al. Understanding Drivers of Resistance Toward Implementation of Web-Based Self-Management Tools in Routine Cancer Care Among Oncology Nurses: Cross-Sectional Survey Study. Journal of Medical Internet Research. 2019;21(12).

39. Woodward EN, Matthieu MM, Uchendu US, Rogal S, Kirchner JE. The health equity implementation framework: proposal and preliminary study of hepatitis C virus treatment. Implementation Science. 2019;14.

40. Lau F, Partridge C, Randhawa G, Bowen M. Applying the Clinical Adoption Framework to Evaluate the Impact of an Ambulatory Electronic Medical Record. Enabling Health and Healthcare through Ict: Available, Tailored and Closer. 2013;183:15-20.

41. Bergstrom A, Skeen S, Duc DM, Blandon EZ, Estabrooks C, Gustavsson P, et al. Health system context and implementation of evidence-based practices-development and validation of the Context Assessment for Community Health (COACH) tool for low- and middle-income settings. Implementation Science. 2015;10.

42. Helfrich CD, Weiner BJ, McKinney MM, Minasian L. Determinants of implementation effectiveness - Adapting a framework for complex innovations. Medical Care Research and Review. 2007;64(3):279-303.

43. Feldstein AC, Glasgow RE. A Practical, Robust Implementation and Sustainability Model (PRISM) for Integrating Research Findings into Practice. Joint Commission journal on quality and patient safety. 2008;34(4):228-43.

44. Pfadenhauer LM, Gerhardus A, Mozygemba K, Lysdahl KB, Booth A, Hofmann B, et al. Making sense of complexity in context and implementation: the Context and Implementation of Complex Interventions (CICI) framework. Implementation Science. 2017;12.

45. Holden RJ, Karsh BT. The Technology Acceptance Model: Its past and its future in health care. Journal of Biomedical Informatics. 2010;43(1):159-72.

46. Blase KA, Van Dyke M, Fixsen DL, Bailey FW. Implementation Science Key Concepts, Themes, and Evidence for Practitioners in Educational Psychology. Handbook of Implementation Science for Psychology in Education. 2012:13-34.

47. Presseau J, McCleary N, Lorencatto F, Patey AM, Grimshaw JM, Francis JJ. Action, actor, context, target, time (AACTT): a framework for specifying behaviour. Implementation Science. 2019;14(1).

48. Weiner BJ. A theory of organizational readiness for change. Implementation Science. 2009;4.

49. Pettigrew J, Segrott J, Ray CD, Littlecott H. Social Interface Model: Theorizing Ecological Post-Delivery Processes for Intervention Effects. Prev Sci. 2018;19(8):987-96.

50. Grol R, Wensing M. What drives change? Barriers to and incentives for achieving evidence-based practice. Medical Journal of Australia. 2004;180(6):S57-S60.

51. Atun R, de Jongh T, Secci F, Ohiri K, Adeyi O. Integration of targeted health interventions into health systems: a conceptual framework for analysis. Health policy and planning. 2010;25(2):104-11.

52. Shaw J, Agarwal P, Desveaux L, Palma DC, Stamenova V, Jamieson T, et al. Beyond "implementation": digital health innovation and service design. NPJ digital medicine. 2018;1.

53. Opoku D, Busse R, Quentin W. Achieving Sustainability and Scale-Up of Mobile Health Noncommunicable Disease Interventions in Sub-Saharan Africa: Views of Policy Makers in Ghana. JMIR mHealth and uHealth. 2019;7(5).

54. Chambers DA, Glasgow RE, Stange KC. The dynamic sustainability framework: addressing the paradox of sustainment amid ongoing change. Implementation Science. 2013;8.

55. Linsky A, Gellad WF, Linder JA, Friedberg MW. Advancing the Science of Deprescribing: A Novel Comprehensive Conceptual Framework. Journal of the American Geriatrics Society. 2019;67(10):2018-22.

56. Cochrane LJ, Olson CA, Murray S, Dupuis M, Tooman T, Hayes S. Gaps between knowing and doing: Understanding and assessing the barriers to optimal health care. J Contin Educ Health Prof. 2007;27(2):94-102.

57. Gurses AP, Marsteller JA, Ozok AA, Xiao Y, Owens S, Pronovost PJ. Using an interdisciplinary approach to identify factors that affect clinicians' compliance with evidence-based guidelines. Critical Care Medicine. 2010;38(8):S282-S91.

58. Durlak JA, DuPre EP. Implementation matters: A review of research on the influence of implementation on program outcomes and the factors affecting implementation. American Journal of Community Psychology. 2008;41(3-4):327-50.

59. Jacobson N, Butterill D, Goering P. Development of a framework for knowledge translation: understanding user context. Journal of health services research & policy. 2003;8(2):94-9.

60. Landry R, Amara N, Lamari M. Climbing the ladder of research utilization - Evidence from social science research. Sci Commun. 2001;22(4):396-422.

61. Ferlie EB, Shortell SM. Improving the quality of health care in the United Kingdom and the United States: A framework for change. Milbank Quarterly. 2001;79(2):281-+.

62. 张寅升, 李昊旻, 段会龙. 面向循证医学知识转化的相关概念及研究现状. 中国全科医学. 2016;19(19):2358.

63. 陈耀龙, 史乾灵, 赵俊强, 徐东, 李慧, 邵瑞太, et al. 从知到行: 跨越指南理论与实践的鸿沟. 协和医学杂志. 2020;11(6):746-53.

64. Moullin JC, Sabater-Hernandez D, Benrimoj SI. Model for the evaluation of implementation programs and professional pharmacy services. Research in Social & Administrative Pharmacy. 2016;12(3):515-22.

65. Stirman SW, Miller CJ, Toder K, Calloway A. Development of a framework and coding system for modifications and adaptations of evidence-based interventions. Implementation Science. 2013;8.

66. Stirman SW, Baumann AA, Miller CJ. The FRAME: an expanded framework for reporting adaptations and modifications to evidence-based interventions. Implementation Science. 2019;14.

67. Glasgow RE, Vogt TM, Boles SM. Evaluating the public health impact of health promotion interventions: The RE-AIM framework. American journal of public health. 1999;89(9):1322-7.

68. Katz J, Wandersman A, Goodman RM, Griffin S, Wilson DK, Schillaci M. Updating the FORECAST formative evaluation approach and some implications for ameliorating theory failure, implementation failure, and evaluation failure. Evaluation and program planning. 2013;39:42-50.

69. Holbrook AM, Hunt SR, See MR. Implementation of Dialectical Behavior Therapy in Residential Treatment Programs: A Process Evaluation Model for a Community-Based Agency. Community Ment Health J. 2018;54(7):921-9.

70. Yusof MM, Kuljis J, Papazafeiropoulou A, Stergioulas LK. An evaluation framework for Health Information Systems: human, organization and technology-fit factors (HOT-fit). Int J Med Inf. 2008;77(6):386-98.

71. Proctor E, Silmere H, Raghavan R, Hovmand P, Aarons G, Bunger A, et al. Outcomes for Implementation Research: Conceptual Distinctions, Measurement Challenges, and Research Agenda. Administration and Policy in Mental Health and Mental Health Services Research. 2011;38(2):65-76.

72. Braganza MZ, Kilbourne AM. The Quality Enhancement Research Initiative (QUERI) Impact Framework: Measuring the Real-world Impact of Implementation Science. Journal of general internal medicine. 2021;36(2):396-403.

73. Ko LK, Jang SH, Friedman DB, Glanz K, Leeman J, Hannon PA, et al. An application of the Science Impact Framework to the Cancer Prevention and Control Research Network from 2014-2018. Preventive medicine. 2019;129.

74. Soi C, Shearer JC, Budden A, Carnahan E, Salisbury N, Asiimwe G, et al. How to evaluate the implementation of complex health programmes in low-income settings: the approach of the Gavi Full Country Evaluations. Health policy and planning. 2020;35:35-46.

75. Chamberlain P, Brown CH, Saldana L. Observational measure of implementation progress in community based settings: The Stages of implementation completion (SIC). Implementation Science. 2011;6.

76. Carroll C, Patterson M, Wood S, Booth A, Rick J, Balain S. A conceptual framework for implementation fidelity. Implementation Science. 2007;2.

77. Dunst CJ, Trivette CM, Raab M. An Implementation Science Framework for Conceptualizing and Operationalizing Fidelity in Early Childhood Intervention Studies. Journal of Early Intervention. 2013;35(2):85-101.

78. Schwarz UV, Aarons GA, Hasson H. The Value Equation: Three complementary propositions for reconciling fidelity and adaptation in evidence-based practice implementation. BMC health services research. 2019;19(1).

79. Kirk MA, Moore JE, Stirman SW, Birken SA. Towards a comprehensive model for understanding adaptations' impact: the model for adaptation design and impact (MADI). Implementation Science. 2020;15(1).

80. Dymoke-Bradshaw K, Brown A. A Reflective Framework for Successful IS Implementation in Healthcare: Applying Theories From IS Innovation and Implementation Research. Proceedings of the 5th European Conference on Information Management and Evaluation. 2011:140-7.

81. Meissner P. LADDERS: A dynamic paradigm for planning, implementing, and evaluating sustainable change in learning health systems. Learning health systems. 2018;2(3).

82. Lennox L, Maher L, Reed J. Navigating the sustainability landscape: a systematic review of sustainability approaches in healthcare. Implementation science : IS. 2018;13(1):27.

83. Bowman CC, Sobo EJ, Asch SM, Gifford AL, Enhancement HHQ. Measuring persistence of implementation: QUERI Series. Implementation Science. 2008;3.

84. Eboreime EA, Olawepo JO, Banke-Thomas A, Abejirinde IOO, Abimbola S. Appraising and addressing design and implementation failure in global health: A pragmatic framework. Global public health. 2020.

85. Michie S, van Stralen MM, West R. The behaviour change wheel: A new method for characterising and designing behaviour change interventions. Implementation Science. 2011;6.

86. Raghavan R, Bright CL, Shadoin AL. Toward a policy ecology of implementation of evidence-based practices in public mental health settings. Implementation Science. 2008;3.

87. Metzl JM, Hansen H. Structural competency: Theorizing a new medical engagement with stigma and inequality. Social Science & Medicine. 2014;103:126-33.

88. Glisson C, Schoenwald SK. The ARC organizational and community intervention strategy for implementing evidence-based children's mental health treatments. Ment Health Serv Res. 2005;7(4):243-59.

89. Oetzel J, Scott N, Hudson M, Masters-Awatere B, Rarere M, Foote J, et al. Implementation framework for chronic disease intervention effectiveness in Maori and other indigenous communities. Globalization and health. 2017;13.

90. Ramsey AT, Proctor EK, Chambers DA, Garbutt JM, Malone S, Powderly WG, et al. Designing for Accelerated Translation (DART) of Emerging Innovations in Health. J Clin Transl Sci. 2019;3(2-3):53-8.

91. Wandersman A, Chien VH, Katz J. Toward an Evidence-Based System for Innovation Support for Implementing Innovations with Quality: Tools, Training, Technical Assistance, and Quality Assurance/Quality Improvement. American Journal of Community Psychology. 2012;50(3-4):445-59.

92. Gravitt PE, Rositch AF, Jurczuk M, Meza G, Carillo L, Jeronimo J, et al. Integrative Systems Praxis for Implementation Research (INSPIRE): An Implementation Methodology to Facilitate the Global Elimination of Cervical Cancer. Cancer Epidemiology Biomarkers & Prevention. 2020;29(9):1710-9.

93. Leeman J, Baernholdt M, Sandelowski M. Developing a theory-based taxonomy of methods for implementing change in practice. J Adv Nurs. 2007;58(2):191-200.

94. Eweje G, Sajjad A, Nath SD, Kobayashi K. Multi-stakeholder partnerships: a catalyst to achieve sustainable development goals. Marketing Intelligence & Planning. 2021;39(2):186-212.

95. Shea J. Taking Nonprofit Intermediaries Seriously: A Middle-Range Theory for Implementation Research. Public Administration Review. 2011;71(1):57-66.

96. Barki H, Pinsonneault A. A model of organizational integration, implementation effort, and performance. Organization Science. 2005;16(2):165-79.

97. Zangiski MADG, de Lima EP, da Costa SEG. Organizational competence building and development: Contributions to operations management. International Journal of Production Economics. 2013;144(1):76-89.

98. Powell BJ, McMillen JC, Proctor EK, Carpenter CR, Griffey RT, Bunger AC, et al. A Compilation of Strategies for Implementing Clinical Innovations in Health and Mental Health. Medical Care Research and Review. 2012;69(2):123-57.

99. Powell BJ, Waltz TJ, Chinman MJ, Damschroder LJ, Smith JL, Matthieu MM, et al. A refined compilation of implementation strategies: results from the Expert Recommendations for Implementing Change (ERIC) project. Implementation Science. 2015;10.

100. Meyers DC, Katz J, Chien V, Wandersman A, Scaccia JP, Wright A. Practical Implementation Science: Developing and Piloting the Quality Implementation Tool. American Journal of Community Psychology. 2012;50(3-4):481-96.

101. Miller WL, Rubinstein EB, Howard J, Crabtree BF. Shifting Implementation Science Theory to Empower Primary Care Practices. Annals of family medicine. 2019;17(3):250-6.

102. Foster-Fishman PG, Nowell B, Yang HL. Putting the system back into systems change: a framework for understanding and changing organizational and community systems. American Journal of Community Psychology. 2007;39(3-4):197-215.

103. Moulton S, Sandfort JR. The Strategic Action Field Framework for Policy Implementation Research. Policy Studies Journal. 2017;45(1):144-69.

104. Fernandez ME, ten Hoor GA, van Lieshout S, Rodriguez SA, Beidas RS, Parcel G, et al. Implementation Mapping: Using Intervention Mapping to Develop Implementation Strategies. Frontiers in public health. 2019;7.

105. Poot CC, van der Kleij RM, Brakema EA, Vermond D, Williams S, Cragg L, et al. From research to evidence-informed decision making: a systematic approach. Journal of Public Health. 2018;40:I3-I12.

106. Smith JD, Li DH, Rafferty MR. The Implementation Research Logic Model: a method for planning, executing, reporting, and synthesizing implementation projects. Implementation Science. 2020;15(1).

107. Kukafka R, Johnson SB, Linfante A, Allegrante JP. Grounding a new information technology implementation framework in behavioral science: a systematic analysis of the literature on IT use. Journal of Biomedical Informatics. 2003;36(3):218-27.

108. Neta G, Glasgow RE, Carpenter CR, Grimshaw JM, Rabin BA, Fernandez ME, et al. A Framework for Enhancing the Value of Research for Dissemination and Implementation. American journal of public health. 2015;105(1):49-57.

109. van Daele T, van Audenhove C, Hermans D, van den Bergh O, van den Broucke S. Empowerment implementation: enhancing fidelity and adaptation in a psycho-educational intervention. Health Promot Internation. 2014;29(2):212-22.

110. Proctor EK, Landsverk J, Aarons G, Chambers D, Glisson C, Mittman B. Implementation Research in Mental Health Services: an Emerging Science with Conceptual, Methodological, and Training challenges. Administration and Policy in Mental Health and Mental Health Services Research. 2009;36(1):24-34.

111. Bradley EH, Curry LA, Taylor LA, Pallas SW, Talbert-Slagle K, Yuan C, et al. A model for scale up of family health innovations in low-income and middle-income settings: a mixed methods study. BMJ open. 2012;2(4).

112. Bragge P, Grimshaw JM, Lokker C, Colquhoun H, Grp AWW. AIMD - a validated, simplified framework of interventions to promote and integrate evidence into health practices, systems, and policies. BMC medical research methodology. 2017;17.

113. Boothroyd RI, Flint AY, Lapiz AM, Lyons S, Jarboe KL, Aldridge WA. Active involved community partnerships: co-creating implementation infrastructure for getting to and sustaining social impact. Translational behavioral medicine. 2017;7(3):467-77.

114. Shea CM, Jacobs SR, Esserman DA, Bruce K, Weiner BJ. Organizational readiness for implementing change: a psychometric assessment of a new measure. Implementation Science. 2014;9.

115. Grimshaw JM, Patey AM, Kirkham KR, Hall A, Dowling SK, Rodondi N, et al. De-implementing wisely: developing the evidence base to reduce low-value care. BMJ Qual Saf. 2020;29(5):409-17.

116. Meyers DC, Durlak JA, Wandersman A. The Quality Implementation Framework: A Synthesis of Critical Steps in the Implementation Process. American Journal of Community Psychology. 2012;50(3-4):462-80.

117. Moullin JC, Sabater-Hernandez D, Benrimoj SI. Qualitative study on the implementation of professional pharmacy services in Australian community pharmacies using framework analysis. BMC health services research. 2016;16.

118. Brown CG. The Iowa Model of Evidence-Based Practice to Promote Quality Care: An Illustrated Example in Oncology Nursing. Clin J Oncol Nurs. 2014;18(2):157-9.

119. Graham ID, Logan J, Harrison MB, Straus SE, Tetroe J, Caswell W, et al. Lost in knowledge translation: Time for a map? J Contin Educ Health Prof. 2006;26(1):13-24.

120. Metz A, Naoom S, Halle T, Bartley L. An integrated stage-based framework for implementation of early childhood programs and systems (OPRE Research Brief OPRE 2015­-48). Washington, DC: Office of Planning, Research and Evaluation, Administration for Children and Families, U.S. Department of Health and Human Services: Washington, DC: Office of Planning, Research and Evaluation, Administration for Children and Families, U.S. Department of Health and Human Services; 2015.

121. Logan J, Graham ID. Toward a comprehensive interdisciplinary model of health care research use. Sci Commun. 1998;20(2):227-46.

122. Wingood GM, DiClemente RJ. The ADAPT-ITT model - A novel method of adapting evidence-based HIV interventions. Jaids-Journal of Acquired Immune Deficiency Syndromes. 2008;47:S40-S6.

123. Aarons GA, Green AE, Palinkas LA, Self-Brown S, Whitaker DJ, Lutzker JR, et al. Dynamic adaptation process to implement an evidence-based child maltreatment intervention. Implementation Science. 2012;7.

124. Miller CJ, Wiltsey-Stirman S, Baumann AA. Iterative Decision-making for Evaluation of Adaptations (IDEA): A decision tree for balancing adaptation, fidelity, and intervention impact. J Community Psychol. 2020;48(4):1163-77.

125. Eslava-Schmalbach J, Garzon-Orjuela N, Elias V, Reveiz L, Tran N, Langlois EV. Conceptual framework of equity-focused implementation research for health programs (EquIR). International journal for equity in health. 2019;18.

126. Sarma H, D'Este C, Ahmed T, Bossert TJ, Banwell C. Developing a conceptual framework for implementation science to evaluate a nutrition intervention scaled-up in a real-world setting. Public Health Nutrition. 2021;24:S7-S22.

127. Sabir M, Breckman R, Meador R, Wethington E, Reid MC, Pillemer K. The CITRA research-practice consensus-workshop model: Exploring a new method of research translation in aging. The Gerontologist. 2006;46(6):833-9.

128. Grol R. Improving Patient Care The Implementation of Change in Health Care SECOND EDITION Introduction. Improving Patient Care: The Implementation of Change in Health Care, 2nd Edition. 2013:Xiii-Xvii.

129. Knapp H, Anaya HD. Implementation Science in the Real World: A Streamlined Model. J Healthc Qual. 2012;34(6):27-35.

130. Craig P, Dieppe P, Macintyre S, Michie S, Nazareth I, Petticrew M. Developing and evaluating complex interventions: the new Medical Research Council guidance. Brit Med J. 2008;337(7676).

131. Ramaswamy R, Shidhaye R, Nanda S. Making complex interventions work in low resource settings: developing and applying a design focused implementation approach to deliver mental health through primary care in India. International journal of mental health systems. 2018;12.

132. Sogolow ED, Kay LS, Doll LS, Neumann MS, Mezoff JS, Eke AN, et al. Strengthening HIV prevention: Application of a research-to-practice framework. Aids Education and Prevention. 2000;12(5):21-32.

133. Stetler CB. Refinement of the Stetler/Marram model for application of research findings to practice. Nursing outlook. 1994;42(1):15-25.

134. Opacua AId. Guide on spread and sustainability. NHS Scotland: Healthcare Improvement Scotland; 2013.

135. Sisk BA, Mozersky J, Antes AL, DuBois JM. The "Ought-Is" Problem: An Implementation Science Framework for Translating Ethical Norms Into Practice. Am J Bioeth. 2020;20(4):62-70.

136. Barker PM, Reid A, Schall MW. A framework for scaling up health interventions: lessons from large-scale improvement initiatives in Africa. Implementation Science. 2016;11.

137. Lyon AR, Wasse JK, Ludwig K, Zachry M, Bruns EJ, Unutzer J, et al. The Contextualized Technology Adaptation Process (CTAP): Optimizing Health Information Technology to Improve Mental Health Systems. Administration and Policy in Mental Health and Mental Health Services Research. 2016;43(3):394-409.

138. Napoles AM, Stewart AL. Transcreation: an implementation science framework for community-engaged behavioral interventions to reduce health disparities. BMC health services research. 2018;18.

139. Cordova-Pozo K, Hoopes AJ, Cordova F, Vega B, Segura Z, Hagens A. Applying the results based management framework to the CERCA multi-component project in adolescent sexual and reproductive health: a retrospective analysis. Reproductive health. 2018;15.

140. Wilson KM, Brady TJ, Lesesne C, Translation NWG. An Organizing Framework for Translation in Public Health: The Knowledge to Action Framework. Preventing chronic disease. 2011;8(2).

141. Stevens KR. The impact of evidence-based practice in nursing and the next big ideas. Online J Issues Nurs. 2013;18(2):4.

142. Majdzadeh R, Sadighi J, Nejat S, Mahani AS, Gholami J. Knowledge Translation for Research Utilization: Design of a Knowledge Translation Model at Tehran University of Medical Sciences. J Contin Educ Health Prof. 2008;28(4):270-7.

143. Davis SM, Peterson JC, Helfrich CD, Cunningham-Sabo L. Introduction and conceptual model for utilization of prevention research. American journal of preventive medicine. 2007;33(1):S1-S5.

144. Pronovost P, Berenholtz S, Needham D. Translating evidence into practice: a model for large scale knowledge translation. Bmj-British Medical Journal. 2008;337(7676).

145. 胡雁, 周英凤, 朱政, 邢唯杰, 成磊, 顾艳荭, et al. 通过循证护理实践 促进护理知识转化. 护士进修杂志. 2015;30(11):961-3.
